# A Prediction of All‐Inorganic Lead‐Free Halide Perovskites for Photovoltaic Application: Rb_3_Mo_2_Br_9_ and Rb_3_Mo_2_Cl_9_

**DOI:** 10.1002/advs.202407751

**Published:** 2024-10-11

**Authors:** Xinxin Deng, Zhesi Zhang, Zili Zhang, Yunyi Wu, Hongzhou Song, Huanxin Li, Bingcheng Luo

**Affiliations:** ^1^ College of Science China Agricultural University Beijing 100083 China; ^2^ Hunan Red Solar Photoelectricity Science and Technology Co., LTD. National Engineering Research Center of Photovoltaic Equipment (NCPVE) Changsha 410000 China; ^3^ School of Science China University of Geosciences Beijing 100083 China; ^4^ Research Center for Comprehensive Energy Technology CTG Science and Technology Research Institute Beijing 100038 China; ^5^ Institute of Applied Physics and Computational Mathematics Beijing 100094 China; ^6^ Department of Chemistry, Physical & Theoretical Chemistry Laboratory University of Oxford Oxford OX1 3QZ UK; ^7^ Electrochemical Innovation Lab, Department of Chemical Engineering University College London London WC1E 7JE UK

**Keywords:** 2D, first‐principles calculations, halide perovskite, solar cell

## Abstract

Lead‐based organic‐inorganic hybrid perovskites show promise as photovoltaic materials due to their high energy conversion efficiencies. However, concerns regarding lead toxicity and the poor environmental and operational stability of the organic cationic group have limited their widespread application. To address these challenges, the design of all‐inorganic lead‐free halide perovskites offers potential solutions for photovoltaic applications. Here, two layered perovskite derivatives, Rb_3_Mo_2_Cl_9_ and Rb_3_Mo_2_Br_9_, are explored, and their electronic, structural, and photovoltaic properties are analyzed using advanced theoretical calculations. Rb_3_Mo_2_Br_9_ exhibits a suitable direct bandgap of 1.60 eV, making it a promising candidate for use as a light absorber in low‐cost, high‐efficiency solar cells. On the other hand, Rb_3_Mo_2_Cl_9_ demonstrates a wide direct bandgap exceeding 1.70 eV, positioning it as a viable option for use as a top cell in tandem photovoltaic systems alongside silicon. Both materials display ideal optical properties in the visible light region and hold promise as excellent inorganic lead‐free perovskite alternatives.

## Graphical abstract

Lead‐based organic‐inorganic hybrid perovskites have demonstrated great potential as photovoltaic materials due to their high energy conversion efficiencies. However, their application is hindered by concerns over lead toxicity and the environmental and operational instability of the organic cations. Two layered perovskite derivatives, Rb_3_Mo_2_Cl_9_ and Rb_3_Mo_2_Br_9_, are investigated by analyzing their electronic, structural, and photovoltaic properties through theoretical calculations.

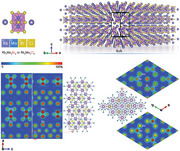

## 1 Introduction

Organic‐inorganic hybrid perovskite solar cells have gained a lot of attention due to their rapidly increasing power conversion efficiency (PCE),^[^ ^1^, ^2^, ^3^, ^4^ ^]^ achieving a record 25.2%^[^ ^5^ ^]^ PCE to date, comparable to that of commercial silicon‐based solar cells.^[^ ^6^, ^7^, ^8^, ^9^ ^]^ For instance, Isikgor et al.^[^ ^10^ ^]^ fabricated MA_1‐x_FA_x_PbI_3‐y_Cl_y_ films that exhibited high power conversion efficiencies as high as 18.14%. Jiang et al.^[^ ^11^ ^]^ reported the organic halide salt phenethyl ammonium iodide (PEAI) on the FA‐MA mixed perovskite film to obtain a planar perovskite solar cell with 23.32% certification efficiency. However, there still exists a gap from the theoretical Shockley‐Queisser limit of 30.5% PCE in a single‐junction cell based on methylammonium lead iodide.^[^ ^12^ ^]^

Despite the rapid advancements in photovoltaic conversion efficiency, numerous technical challenges remain to be addressed.^[^ ^13^, ^14^ ^]^ These include concerns related to lead toxicity and inadequate long‐term stability against moisture and air. A practical approach is to design stable and non‐toxic alternatives to perovskite materials, such as by substituting Pb with other cations, such as Sn, Ge,^[^ ^15^ ^]^ Bi, Sb^[^ ^16^ ^]^ to form lead‐free halide perovskites.^[^ ^17^ ^]^ The element Sn, being homologous to Pb and its nearest neighbor on the periodic table, is of primary consideration. It shares a similar valence electron configuration with Pb but has a smaller radius, lower atomic mass, and a higher 5 s orbital level. These factors enable the formation of a perovskite film with a reduced bandgap, consequently broadening the sunlight absorption spectrum. However, tin‐based perovskites face relative instability due to the ease with which Sn^2+^ is oxidized to compounds containing Sn^4+^, and the inherent defectiveness stemming from Sn vacancies.^[^ ^18^ ^]^ In Bi‐doped MAPbI_3_, the perovskite bandgap is substantially unchanged, and the absorption begins to red shift due to the Bi‐defective state.^[^ ^19^ ^]^ It is found that Bi^3+^ doping can increase the α‐phase stability of CsPbI_3_, thus improving the efficiency of fully inorganic perovskite solar cells.^[^ ^19^ ^]^

Recently, trivalent Bi^3+^ and Sb^3+^‐based compounds A_3_B_2_X_9_^[^ ^16^ ^]^ have been examined as photovoltaic absorbers.^[^ ^20^ ^]^ In this context, a series of inorganic lead‐free perovskites have entered the field of vision, compared with ABX_3_ perovskites,^[^ ^21^, ^22^ ^]^ and a novel inorganic A_3_B_2_X_9_ perovskite has greatly improved stability and reduced toxicity. A_3_B_2_X_9_ is a structurally rich class of perovskite‐like structures,^[^ ^23^ ^]^ where A is a monovalent cation such as Cs, Rb, and K, and B is an inorganic cation such as Bi and Sb. This type of perovskite derivative material has a similar wide bandgap (Eg = 2.0 eV).^[^ ^24^ ^]^ In 2015, Bai et al.^[^ ^25^ ^]^ proposed a dissolution‐recrystallization method to produce Cs_3_Bi_2_I_9_ perovskite nanosheet films, achieving a high power conversion efficiency of 3.20% in solar cells using CuI as a hole transport material. Wei et al.^[^ ^26^ ^]^ synthesized a perovskite material Cs_3_Fe_2_Br_9_ with a defect state, which is black with a low optical bandgap of 1.65 eV and exhibits antiferromagnetic behavior below T_N_ = 13 K. Saparov et al.^[^ ^16^ ^]^ prepared a lead‐free layered perovskite derivative Cs_3_Sb_2_I_9_ films using a two‐step deposition method with a bandgap value of 2.05 eV, and displayed a high absorption level compared to CH_3_NH_3_PbI_3_ and enhanced stability. Although the phase transition of Cs compounds has been well studied, little research has been done on Rb‐based A_3_B_2_X_9_ perovskites. The central symmetric structure of perovskite Rb_3_Bi_2_I_9_ was reported.^[^ ^27^ ^]^ And displayed a bandgap of ≈2 eV by UV–vis and UV emission spectroscopy. However, the VBM level was significantly lower than the widely used lead halide perovskite. Therefore, it is necessary to design a suitable matching energy band for applications in solar cells. Saliba et al.^[^ ^28^ ^]^ introduced oxidized cerium ions (Rb^+^) into perovskite solar cells, which achieved a stable efficiency of 21.6% and operated steadily for 500 h at 85 °C.

We present the all‐inorganic lead‐free perovskite derivatives Rb_3_Mo_2_Cl_9_ and Rb_3_Mo_2_Br_9_ for potential photovoltaic applications. Our findings indicate that Rb_3_Mo_2_Cl_9_ and Rb_3_Mo_2_Br_9_ exhibit bandgaps of 1.78 and 1.60 eV, respectively. Moreover, they demonstrate ideal optical absorption coefficients within the visible light spectrum. Previous studies have revealed that incorporating the small and oxidation‐stable rubidium cation (Rb^+^) into a “cation cascade” can lead to the creation of perovskite materials with exceptional material properties.^[^ ^28^ ^]^ Mo‐containing compounds exhibit relatively good performance, likely due to Mo's possession of d orbitals, which contribute to higher ionic conductivity and stability.^[^ ^29^ ^]^ Here, for the first time, we employ density functional theory to investigate the effects of the perovskite structures and photoelectric properties of Rb_3_Mo_2_Cl_9_ and Rb_3_Mo_2_Br_9_. These materials, serving as optoelectronic components, hold significant promise in advancing perovskite solar cell technology and represent a pivotal step towards the development of lead‐free inorganic perovskite materials.

## 2 Results and Discussion

**Figure 1:**
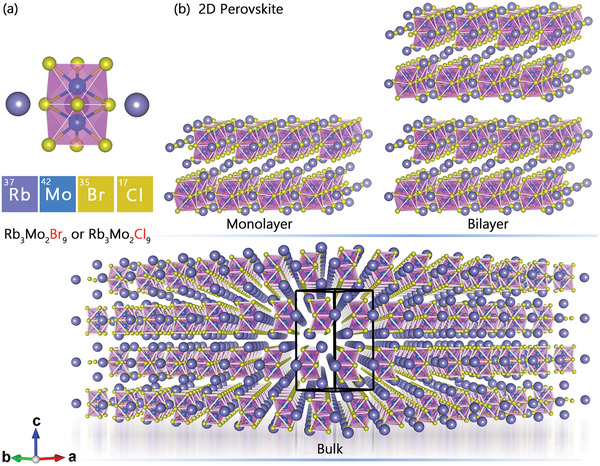
a) Atomic lattice structure of Rb_3_Mo_2_Br_9_ and Rb_3_Mo_2_Cl_9_; b) Monolayer, bilayer, and bulk structures of Rb_3_Mo_2_Br_9_ and Rb_3_Mo_2_Cl_9_.

As depicted in **Figure** **1**, the Cl atoms within the three coplanar planes of each octahedron are shared. The interconnectedness of two adjacent octahedral structures in the geometric arrangement of Rb_3_Mo_2_Cl_9_ crystal cells occurs through the sharing of Cl atoms, resulting in the formation of a 2D spatial arrangement, as illustrated in Figure 1b.

**Figure 2:**
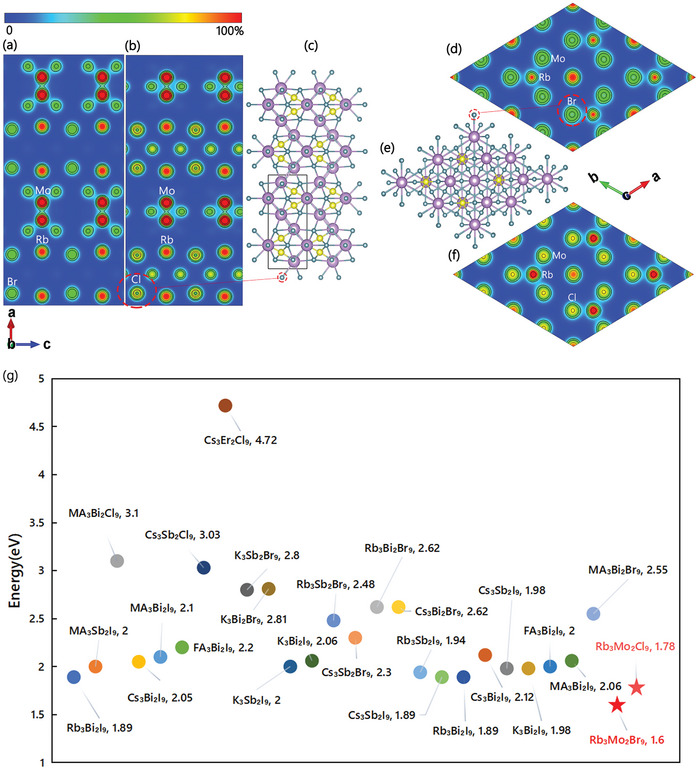
The electron charge density of a) Rb_3_Mo_2_Br_9_ and b)Rb_3_Mo_2_Cl_9_, and c) corresponding lattice structure positions in the (100) direction. Electron charge density of d) Rb_3_Mo_2_Br_9_ and f) Rb_3_Mo_2_Cl_9_, and e) corresponding lattice structure positions in the (001) direction. g) Band alignment diagrams of several common A_3_B_2_X_9_ structures.

The prevalent spatial phenomenon of Rb_3_Mo_2_Cl_9_ observed from the (100) plane perspective underscores its uniform unit arrangement and independent structural integrity, rendering it resistant to fracture. At equilibrium, the atoms exhibit minimal displacement, contributing to the high lattice stability of the compound. The results are consistent with the experimental results of this type of perovskite.^[^ ^30^ ^]^ The lattice constants are a = 7.26, b = 7.26, c = 16.39 Å. The geometric structure of Rb_3_Mo_2_Br_9_ shows the same phenomenon as Rb_3_Mo_2_Cl_9_ in different directions. The lattice constants are a = 7.96, b = 7.96, c = 16.01 Å. Meanwhile, **Figure** **2**a,b shows the charge density of Rb_3_Mo_2_Cl_9_ and Rb_3_Mo_2_Br_9_. The electron charge density of the Cl atom per unit volume is observed to be higher than that of the Br atom (Figure 2c–f).

**Figure 3:**
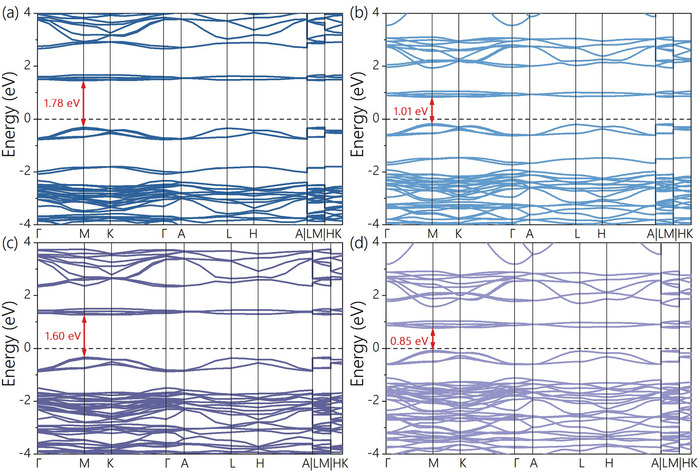
Band structure of a,b) Rb_3_Mo_2_Cl_9_ and c,d) Rb_3_Mo_2_Br_9_. a–c) The HSE calculation; b–d) the PBE calculation.

Figure 2g and Table S2 presents band alignment diagrams for several commonly observed A_3_B_2_X_9_ structures.^[^ ^31^, ^32^, ^33^, ^34^, ^35^, ^36^, ^37^, ^38^, ^39^ ^]^ **Figure** **3** illustrates the band structures of Rb_3_Mo_2_Cl_9_ and Rb_3_Mo_2_Br_9_ in the first Brillouin region along high symmetric K‐points by Heyd‐ Scuseria‐ Ernzerhof (HSE) / Perdew‐Burke‐Ernzerhof (PBE) calculation, with the Fermi level set to zero. The lower computationally expensive PBE functional is well known to underestimate the bandgap. While HSE has a large amount of computation, it is accurate for the calculation of the bandgap.^[^ ^40^, ^41^ ^]^ As depicted in Figure 3b–d, the bandgap width of Rb_3_Mo_2_Cl_9_ is determined to be 1.01 eV through PBE calculation, while that of Rb_3_Mo_2_Br_9_ is measured at 0.85 eV. A more accurate band structure diagram as shown in Figure 3a–c is obtained through HSE recalculation. The HSE calculation shows that the bandgap of Rb_3_Mo_2_Cl_9_ is 1.78 eV, and its valence band maximum (VBM) and conduction band minimum (CBM) are at the same Brillouin region M point, which is a direct bandgap semiconductor. Rb_3_Mo_2_Br_9_ has a bandgap of 1.60 eV, and its VBM and CBM are also at the M point, which is also a direct bandgap semiconductor. They will be widely used in photoelectric applications. For instance, a wide‐gap perovskite material with a bandgap of above 1.70 eV can be widely used in tandem cells with Si, CIGS, or low‐band gap perovskites.^[^ ^42^, ^43^ ^]^ The 1.60 eV bandgap perovskites are ideal photovoltaic materials for multiple fronts.^[^ ^44^ ^]^

**Figure 4:**
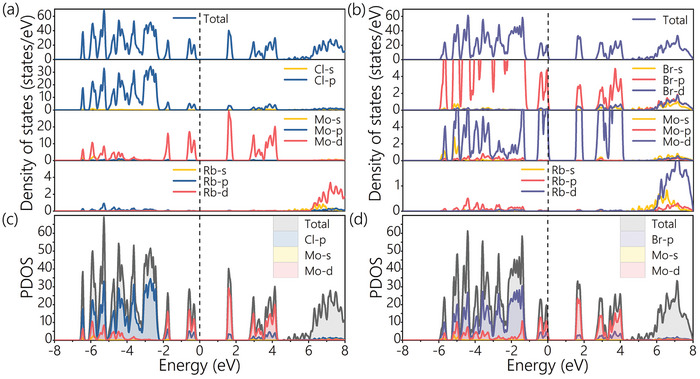
Total density of states and partial density of states of a–c) Rb_3_Mo_2_Cl_9_ and b–d) Rb_3_Mo_2_Br_9_.

The density of states, as another representation of energy band structure, offers a more intuitive insight into the contribution of each orbital to the energy band from an energy perspective. The density of states as shown in **Figure** **4** is obtained through HSE06 calculation and Figure S1 is obtained through PBE calculation. The density of states of Rb_3_Mo_2_Cl_9_ near the Fermi level is mainly contributed by d state of Mo atoms and the p state of Cl atoms, and results are shown in Figure 4a. The density of states of Rb_3_Mo_2_Br_9_ at the Fermi level is mainly contributed by d state of Mo atoms and the p and d states of Br atoms, and results are shown in Figure 4b. The partial density of states (PDOS) can analyze the valence electron quantum states and their orbits better than the density of states. In Figure 4c, the VBM of Rb_3_Mo_2_Cl_9_ primarily arises from Mo‐d, Mo‐s, and Cl‐p orbitals, while the CBM is predominantly contributed by Mo‐d and Cl‐p orbitals. Similarly, in Figure 4d, the VBM of Rb_3_Mo_2_Br_9_ is mainly influenced by Mo‐d, Mo‐s, and Br‐p orbitals, with the CBM primarily contributed by Mo‐d and Br‐p orbitals. Analysis of the density of states reveals that Mo atoms significantly influence their orbitals, particularly the d orbitals in Rb_3_Mo_2_Cl_9_. The presence of d orbitals in Mo can contribute to higher ionic conductivity and stability, which is beneficial for the performance of perovskite materials. For future work, we plan to further research on ion conductivity and its implications for the performance of Rb_3_Mo_2_Cl_9_ and Rb_3_Mo_2_Br_9_. Conversely, Rb exhibits minimal contribution to the states surrounding both CBM and VBM in both materials.

**Figure 5:**
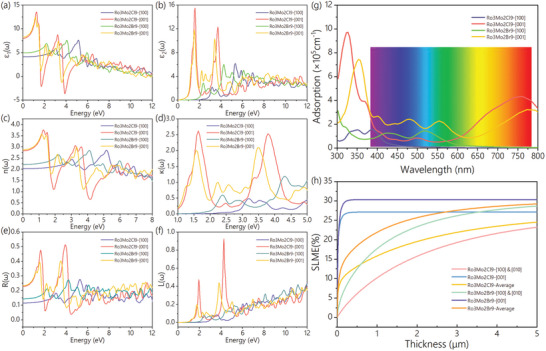
a) Real and b) imaginary parts of the dielectric function, c) refractive index n(ω), d) extinction coefficient κ(ω), e) reflection function R(ω), f) loss function L(ω), g) absorption coefficient of Rb_3_Mo_2_Cl_9_ and Rb_3_Mo_2_Br_9_ in the [100] and [001] directions. h) The SLME is a function of film thickness for Rb_3_Mo_2_Cl_9_ and Rb_3_Mo_2_Br_9_.

The optical properties of these two perovskite derivatives were investigated by obtaining their dielectric functions. The absorption coefficient (*a)*, refractive index (*n)*, extinction coefficient (*κ)*, reflection function (*R)*, and loss function (*L)* are calculated and analyzed. As shown in **Figure** **5**a,b, both the real and imaginary parts of the dielectric functions of Rb_3_Mo_2_Cl_9_ and Rb_3_Mo_2_Br_9_ show strong anisotropy in the directions of [100] and [001]. In the direction of [001], the real and imaginary parts of their dielectric functions have similar changing trends, and the maximum values are all located in the infrared band. The peak values of the imaginary part of Rb_3_Mo_2_Cl_9_ and Rb_3_Mo_2_Br_9_ are 15.54 and 11.87 respectively. At the direction of [100], their imaginary part peaks of Rb_3_Mo_2_Cl_9_ and RB_3_Mo_2_Br_9_ shift to the higher frequency near‐ultraviolet band. The real part of the static dielectric function at the zero‐frequency limit in the [001] direction is higher than that in the [100] direction. The value of Rb_3_Mo_2_Cl_9_ is 8.01, and the value of RB_3_Mo_2_Br_9_ is 8.28. As shown in Figure 5g, the peak absorption coefficients of Rb_3_Mo_2_Cl_9_ and Rb_3_Mo_2_Br_9_ appear in the ultraviolet region. In the direction of [001], the absorption coefficient of Rb_3_Mo_2_Cl_9_ in the visible light region increases until 761 nm, while the rising trend of Rb_3_Mo_2_Br_9_ is not as obvious as that of Rb_3_Mo_2_Cl_9_, and it has a large peak at 772 nm. In the direction of [100], the absorption coefficient of Rb_3_Mo_2_Cl_9_ decreases from ≈400 nm and that of Rb_3_Mo_2_Br_9_ decreases from ≈530 nm.

As shown in Figure 5c, the refractive index of Rb_3_Mo_2_Cl_9_ shows a minimum value ≈of 4 eV, while the absorption coefficient peaks ≈at 320 nm. In other words, the decrease of the refractive index has a positive effect on the absorption coefficient. The same conclusion is also shown for Rb_3_Mo_2_Br_9_. Meanwhile, the light absorption coefficient is proportional to the extinction coefficient. As shown in Figure 5d, the extinction coefficients of Rb_3_Mo_2_Cl_9_ and Rb_3_Mo_2_Br_9_ both show this phenomenon. The extinction coefficient peaks in the visible region along the [001] direction. The reflection function of Rb_3_Mo_2_Cl_9_ is shown in Figure 5e, in which the maximum front appears at 3.92 eV in the direction of [001], and a similar secondary front appears at 1.63 eV in the visible region. The refraction function of Rb_3_Mo_2_Br_9_ is also demonstrated in Figure 5e. The maximum front appears at 3.50 eV in the direction of [001], and the secondary front appears at 1.56 eV in the visible region, which is approximately equal to the peak value of the main peak. The loss functions of Rb_3_Mo_2_Cl_9_ and Rb_3_Mo_2_Br_9_ are shown in Figure 5f. In the visible light region at the direction of [100], the loss functions for both are small, and the maximum is less than 1. The peak appears in the direction of [001] but is relatively small. Both materials have excellent photoelectric properties, especially Rb_3_Mo_2_Cl_9_.

The photovoltaic characteristics of Rb_3_Mo_2_Cl_9_ and Rb_3_Mo_2_Br_9_ were estimated using the Solar light‐to‐electricity conversion efficiency (SLME) method proposed by Yu and Zunger.^[^ ^45^ ^]^ The SLME method utilizes thermodynamic methods and takes into account the specific absorption spectrum and electron‐hole recombination process, which affect the photovoltaic conversion efficiency, to obtain a photovoltaic conversion efficiency that is more in line with reality. The SLME of both materials display changes with the film thickness are shown in Figure 5h. The SLME first increases rapidly with the increase of film thickness, then reaches a peak and stabilizes. The maximum SLME value of Rb_3_Mo_2_Br_9_ along the [001] direction can reach 30.35%, and the maximum value of Rb_3_Mo_2_Cl_9_ can reach 27.14%, which promises to achieve extremely high photovoltaic efficiency.

## 3 Conclusion

In conclusion, our first‐principles calculations have probed the electronic, structural, and optical characteristics of the lead‐free perovskites Rb_3_Mo_2_Cl_9_ and Rb_3_Mo_2_Br_9_. Our results indicate that the integration of molybdenum (Mo) into these compounds significantly boosts their structural robustness. The bandgap analysis shows that Rb_3_Mo_2_Cl_9_ has a bandgap of 1.78 eV, positioning it as a complementary material for use in tandem with low‐bandgap solar cells. In contrast, Rb_3_Mo_2_Br_9_ possesses a bandgap of 1.60 eV, marking it as a highly suitable candidate for direct application in photovoltaic devices. The density of states calculations highlight the substantial contribution of Mo atoms to the electronic orbitals of both materials, with the Mo‐d orbitals playing a particularly pivotal role in the case of Rb_3_Mo_2_Cl_9_. In contrast, the contribution from rubidium (Rb) atoms was found to be negligible. As for their optical properties, both Rb_3_Mo_2_Cl_9_ and Rb_3_Mo_2_Br_9_ exhibit robust light absorption across the visible and ultraviolet spectra, with Rb_3_Mo_2_Cl_9_ demonstrating an even more favorable absorption coefficient. SLME analyses predict remarkable photovoltaic efficiencies of 30.35% for Rb_3_Mo_2_Br_9_ and 27.14% for Rb_3_Mo_2_Cl_9_, underscoring their potential for high‐performance solar energy conversion. By adopting a lead‐free approach in the design of these perovskites, we aim to mitigate environmental pollution, thereby broadening their applicability in sustainable technologies. This study not only demonstrates the promising photovoltaic performance of these lead‐free compounds but also provides a critical foundation for the ongoing development of lead‐free inorganic perovskites, offering a pathway toward more sustainable and environmentally friendly solar cell technologies.

## 4 Experimental Section

All‐inorganic lead‐free perovskite derivatives Rb_3_Mo_2_Cl_9_ and Rb_3_Mo_2_Br_9_ were studied using density functional theory (DFT) based first‐principles calculations (Table S1). Structural, electronic, and optical properties were carried out through the VASP program.^[^ ^46^, ^47^ ^]^ Generalized gradient approximation (GGA) with the exchange and correlation in the PBE formalisms were used to optimize the geometric structure and perform the self‐consistent field calculations. Within the converge tests, a cut‐off energy of 600 eV and a Monkhorst‐Pack mesh grid of 6 × 6 × 2 were employed.^[^ ^48^ ^]^ The energy tolerance and the force tolerance were set as 1 × 10^−8 ^eV per atom and 0.001 eV Å^−1^, respectively. HSE06 hybrid functional were employed to achieve accurate band structure.^[^ ^49^, ^50^ ^]^ The pseudopotentials were constructed by the electron configurations as Rb 4s24p^6^5s^1^ states, Cl 3s23p^5^ states, Br 4s24p^5^ states, and Mo 4s24p^6^4d^5^5s^1^ states (Supporting Information).

The following physical parameter methods are used in the study of optical properties. The physical meaning of the dielectric function (*ε*) is the resistance of the material to the external field, which is defined as

(1) $$\varepsilon \left(\omega \right)=\varepsilon_{1}\left(\omega \right)+i\varepsilon_{2}\left(\omega \right)$$

where *ω* is frequency, *ε_1_* is the real part, *ε_2_* is the imaginary part. The *ε_1_* can modulate the phase of the function to represent the dispersion. The *ε_2_* can modulate the amplitude of the function to show changes in gain or loss. The *a* can be calculated from the refractive index.

(2) $$a\;\left(\omega \right)=\frac{2\varepsilon_{2}\omega }{n\left(\omega \right)c}$$

where *c* is the speed of light in a vacuum. The *n* is expressed as follows.

(3) $$n\left(\omega \right)=\sqrt{\frac{\sqrt{\varepsilon_{1}^{2}+\varepsilon_{2}^{2}}+\varepsilon_{1}}{2}}$$

At the same time, the *κ* and *L* can be calculated from the real and imaginary parts of the dielectric function. The *R* can be obtained by refractive index and extinction coefficient.

(4) $$\kappa \left(\omega \right)=\sqrt{\frac{\sqrt{{\varepsilon_{1}}^{2}+{\varepsilon_{2}}^{2}}-\varepsilon_{1}}{2}}$$

(5) $$L\left(\omega \right)=\frac{\varepsilon_{2}\left(\omega \right)}{{\varepsilon_{1}}^{2}\left(\omega \right)+{\varepsilon_{2}}^{2}\left(\omega \right)}$$

(6) $$R\left(\omega \right)=\frac{{\left(n(\omega )-1\right)}^{2}+\kappa {\left(\omega \right)}^{2}}{{\left(n(\omega )+1\right)}^{2}+\kappa {\left(\omega \right)}^{2}}$$

## Conflict of Interest

The authors declare no conflict of interest.

## Supporting information



Supporting Information

## Data Availability

The data that support the findings of this study are available from the corresponding author upon reasonable request.
