# Supplementary material for: A Prediction of All‐Inorganic Lead‐Free Halide Perovskites for Photovoltaic Application: Rb3Mo2Br9 and Rb3Mo2Cl9
Source: Adv Sci (Weinh). 2024 Oct 11;11(45):2407751. doi: 10.1002/advs.202407751 (PMC11615774; doi:10.1002/advs.202407751)

**Supporting Information**

A prediction of all-Inorganic lead-free Halide Perovskites for Photovoltaic Application: Rb_3_Mo_2_Br_9_ and Rb_3_Mo_2_Cl_9_

Xinxin Deng^1,2,3^, Zhesi Zhang^1^, Zili Zhang^3^, Yunyi Wu^4^, Hongzhou Song^5^, Huanxin Li^6,7*^, Bingcheng Luo^1*^

^1^ College of Science, China Agricultural University, Beijing 100083, China. Email: [luobc21@cau.edu.cn](mailto:luobc21@cau.edu.cn)

^2^ Hunan Red Solar Photoelectricity Science and Technology Co., LTD. National Engineering Research Center of Photovoltaic Equipment (NCPVE) , Changsha, 410000, China

^3^ School of Science, China University of Geosciences, Beijing 100083, China

^4^ Research Center for Comprehensive Energy Technology, CTG Science and Technology Research Institute, Beijing 100038, China

^5^ Institute of Applied Physics and Computational Mathematics, Beijing 100094, China

^6^ Department of Chemistry, Physical & Theoretical Chemistry Laboratory, University of Oxford, Oxford OX1 3QZ, United Kingdom.

^7^ Electrochemical Innovation Lab, Department of Chemical Engineering, University College London, London, WC1E 7JE, United Kingdom. Email: [huanxin.li@ucl.ac.uk](mailto:huanxin.li@chem.ox.ac.uk)

Table S1. The Direct coordinates of the atomic lattice structure of Rb_3_Mo_2_Br_9_ and Rb_3_Mo_2_Cl_9_.

| **Rb_3_Mo_2_Br_9_** | | |
| --- | --- | --- |
| 0.0000012472944135 | 0.9999987404376043 | 0.2500007239916755 |
| 0.9999987987879422 | 0.0000012082691612 | 0.7499994793525104 |
| 0.3333331738490486 | 0.6666729511974339 | 0.0687539369723567 |
| 0.6666676311599105 | 0.3333262138047104 | 0.9312467266971680 |
| 0.6666742262611081 | 0.3333318865948343 | 0.5687540628043593 |
| 0.3333257212944574 | 0.6666681502420317 | 0.4312455305792326 |
| 0.3333417186512264 | 0.6666608544915036 | 0.8185240885133283 |
| 0.6666584095811103 | 0.3333390186545202 | 0.1814760557186759 |
| 0.6666610380331761 | 0.3333414715260545 | 0.3185276719595365 |
| 0.3333386716837055 | 0.6666588293069324 | 0.6814726151430648 |
| 0.4898500719349812 | 0.9797306606531606 | 0.2500005325440284 |
| 0.5101495009337711 | 0.0202691572925247 | 0.7499995191155904 |
| 0.0202693096599162 | 0.5101499065080901 | 0.2499990906490552 |
| 0.9797308803007638 | 0.4898503660433278 | 0.7500009562169581 |
| 0.4898546949614797 | 0.5101452530427437 | 0.2499997517191872 |
| 0.5101451446753913 | 0.4898550809361240 | 0.7499996943826304 |
| 0.8253815117648244 | 0.6507894880902114 | 0.0958905643367345 |
| 0.1746187962708490 | 0.3492096379981788 | 0.9041086732630532 |
| 0.3492055289344123 | 0.1746130943152622 | 0.0958913225909370 |
| 0.6507953362395611 | 0.8253867181171799 | 0.9041079213022485 |
| 0.8253858445583049 | 0.1746083910331180 | 0.0958952659296202 |
| 0.1746142159510526 | 0.8253914113932836 | 0.9041043861799238 |
| 0.1746134140413602 | 0.3492082644723880 | 0.5958931155548299 |
| 0.8253857245757246 | 0.6507916360314070 | 0.4041073007169729 |
| 0.6507868259263319 | 0.8253814256224388 | 0.5958923544606307 |
| 0.3492132827016334 | 0.1746195764943721 | 0.4041080612059531 |
| 0.1746087953842235 | 0.8253853912577398 | 0.5958972228736030 |
| 0.8253904845894411 | 0.1746152161734287 | 0.4041033752259580 |
| **Rb_3_Mo_2_Cl_9_** | | |
| 0.0000040715103182 | 0.9999959492286621 | 0.2499917403396168 |
| 0.9999958881694653 | 0.0000040917372885 | 0.7500078077334607 |
| 0.3333316941767848 | 0.6666679157897164 | 0.0727886822301471 |
| 0.6666680743669957 | 0.3333322557002347 | 0.9272106451605282 |
| 0.6666675219756826 | 0.3333321478639846 | 0.5727856161533609 |
| 0.3333323105687995 | 0.6666680992743395 | 0.4272138098781042 |
| 0.3333288509644703 | 0.6666709007070750 | 0.8211442726717095 |
| 0.6666712016111163 | 0.3333290245162246 | 0.1788554892145484 |
| 0.6666708781419572 | 0.3333289524663741 | 0.3211327031517186 |
| 0.3333291464562436 | 0.6666710424488471 | 0.6788682714323713 |
| 0.4922404734050616 | 0.9844694364751376 | 0.2499930340569776 |
| 0.5077594817120641 | 0.0155296409929662 | 0.7500068683673149 |
| 0.0155305842346891 | 0.5077596988569582 | 0.2499931050232505 |
| 0.9844703385080678 | 0.4922407284322929 | 0.7500067799134484 |
| 0.4922405236904766 | 0.5077593468337298 | 0.2499929933083962 |
| 0.5077588349259159 | 0.4922409002020771 | 0.7500069091531216 |
| 0.8237127807718636 | 0.6474142856868497 | 0.0967409313053551 |
| 0.1762871414575073 | 0.3525858073166432 | 0.9032593288679820 |
| 0.3525860109313115 | 0.1762874372335688 | 0.0967408769391511 |
| 0.6474138480974716 | 0.8237122632428182 | 0.9032593947203935 |
| 0.8237141433489938 | 0.1762863833026671 | 0.0967408506136280 |
| 0.1762860879325956 | 0.8237136466545181 | 0.9032593537410136 |
| 0.1762884379790606 | 0.3525870524580981 | 0.5967538345066714 |
| 0.8237107497619007 | 0.6474114456784150 | 0.4032463382231839 |
| 0.6474132420631449 | 0.8237117065751960 | 0.5967539039045278 |
| 0.3525883081503522 | 0.1762888362205857 | 0.4032462805529207 |
| 0.1762872634104085 | 0.8237133340120337 | 0.5967537158913103 |
| 0.8237121116772883 | 0.1762876700927265 | 0.4032464629457593 |

Table S2. Band alignment diagrams of several common A_3_B_2_X_9_ structures.

| **A_3_B_2_X_9_** | **Band (eV)** |
| --- | --- |
| Rb₃Bi₂I₉ | 1.89^[34]^ |
| MA₃Sb₂I₉ | 2^[35]^ |
| MA₃Bi₂Cl₉ | 3.1^[35]^ |
| Cs₃Bi₂I₉ | 2.05^[37]^ |
| MA₃Bi₂I₉ | 2.1^[35]^ |
| FA₃Bi₂I₉ | 2.2^[35]^ |
| Cs₃Sb₂Cl₉ | 3.03^[36]^ |
| Cs₃Er₂Cl₉ | 4.72^[36]^ |
| K₃Sb₂Br₉ | 2.8^[38]^ |
| K₃Bi₂Br₉ | 2.81^[38]^ |
| K₃Sb₂I₉ | 2^[38]^ |
| K₃Bi₂I₉ | 2.06^[38]^ |
| Rb₃Sb₂Br₉ | 2.48^[38]^ |
| Cs₃Sb₂Br₉ | 2.3^[38]^ |
| Rb₃Bi₂Br₉ | 2.62^[38]^ |
| Cs₃Bi₂Br₉ | 2.62^[39]^ |
| Rb₃Sb₂I₉ | 1.94^[39]^ |
| Cs₃Sb₂I₉ | 1.89^[39]^ |
| Rb₃Bi₂I₉ | 1.89^[39]^ |
| Cs₃Bi₂I₉ | 2.12^[40]^ |
| Cs₃Sb₂I₉ | 1.98^[40]^ |
| K₃Bi₂I₉ | 1.98^[41]^ |
| FA₃Bi₂I₉ | 2^[41]^ |
| MA₃Bi₂I₉ | 2.06^[42]^ |
| MA₃Bi₂Br₉ | 2.55^[42]^ |
| Rb₃Mo₂Br₉ | 1.6 |
| Rb₃Mo₂Cl₉ | 1.78 |

*The citation number corresponds to the text.

Figure S1. Total density of states and partial density of states of (a, c) Rb_3_Mo_2_Cl_9_ and (b, d) Rb_3_Mo_2_Br_9_ obtained by PBE calculation.


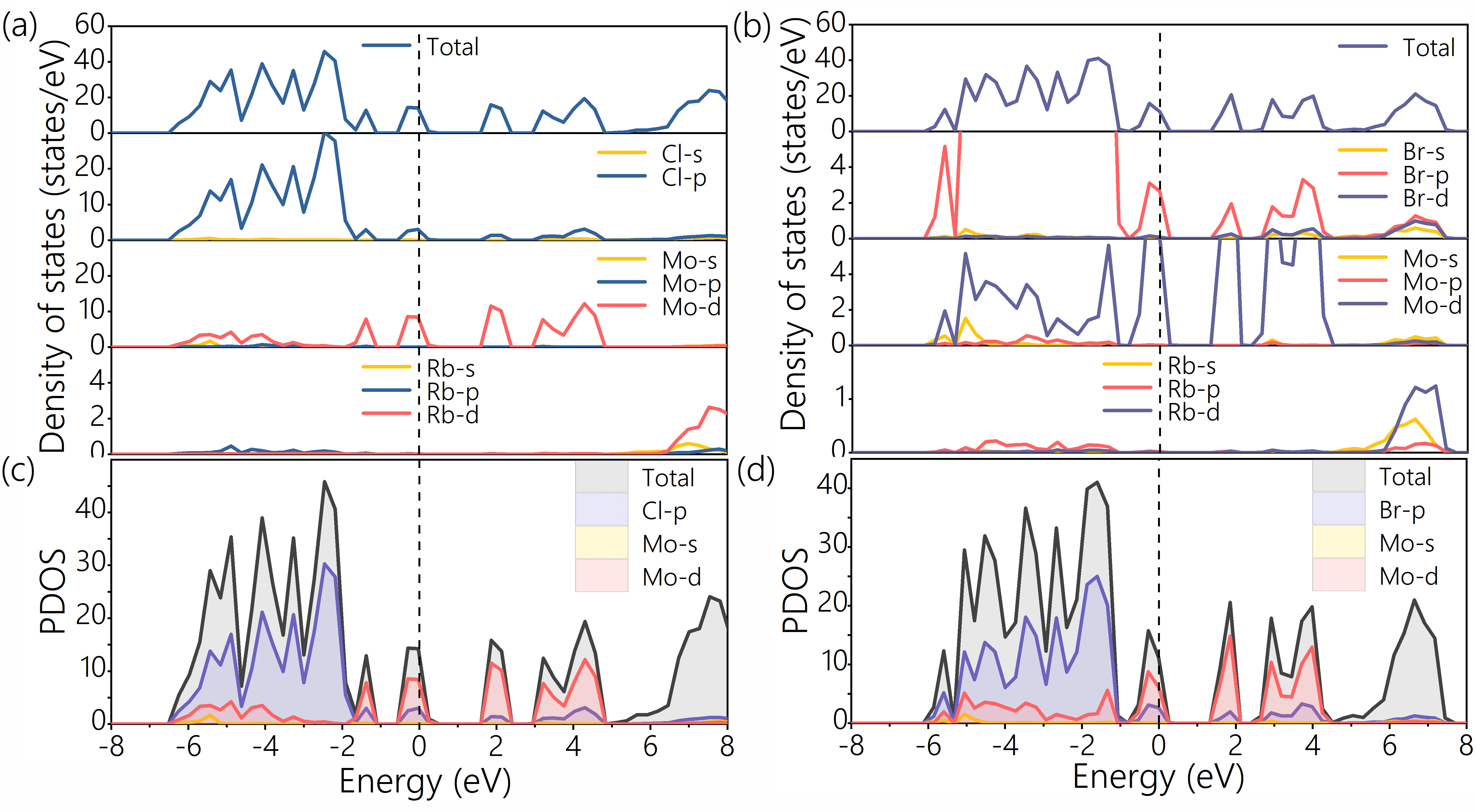

Supplement: Supplementary file 1 — Supporting Information [file ADVS-11-2407751-s001.docx]
